# Production of Green Synthesized Zinc Oxide Nanoparticle-Reinforced PMMA-Based Photopolymer Resins on DLP-Based 3D Printers and Characterization

**DOI:** 10.3390/polym18101229

**Published:** 2026-05-18

**Authors:** Behiç Selman Erdoğdu, Muhammed İhsan Özgün, Emrah Madenci, Mehmet Ali Sayınbatur, Fatih Erci

**Affiliations:** 1 Department of Molecular Biology and Genetics, Faculty of Science, Necmettin Erbakan University, Konya 42090, Türkiye; selmanerdogdu@gmail.com; 2 Department of Metallurgy and Material Engineering, Faculty of Engineering, Necmettin Erbakan University, Konya 42090, Türkiye; miozgun@erbakan.edu.tr; 3 Science and Technology Research and Application Center (BITAM), Necmettin Erbakan University, Konya 42090, Türkiye; 4 Department of Civil Engineering, Necmettin Erbakan University, Konya 42090, Türkiye; 5 Department of Technical Sciences, Western Caspian University, Baku 1001, Azerbaijan; 6 Department of Biotechnology, Graduate School of Natural and Applied Sciences, Necmettin Erbakan University, Konya 42090, Türkiye; m.alisayinbatur@gmail.com; 7 Department of Biotechnology, Faculty of Science, Necmettin Erbakan University, Konya 42090, Türkiye

**Keywords:** 3D printing, phyto-mediated synthesis, ZnO nanoparticles, nanocomposite, photopolymer resin, mechanical properties

## Abstract

In this study, the structural, thermal, and mechanical properties of nanocomposites obtained by adding zinc oxide (ZnO) nanoparticles (NPs), produced by phyto-mediated synthesis using *Dianthus chinensis* plant extract, to a PMMA-based photopolymer resin at different ratios (0.05%, 0.10%, 0.15%, 0.20%, and 0.25%, by weight) were evaluated. The prepared composite resins were produced in different test geometries using a DLP (digital light processing)-based 3D printer (Asiga Ultra). Following the structural characterization of ZnO nanoparticles, tensile, compressive, and flexural mechanical tests were performed on the resulting composites, as well as FTIR, TGA, DSC, and DMA analyses. The FTIR results showed that ZnO NPs were physically integrated into the matrix. TGA and DSC analyses revealed that the addition of ZnO NPs, particularly at an addition rate of 0.15%, increased thermal stability. DMA analyses showed an increase in storage modulus and glass transition temperature as the addition rate increased. In mechanical tests, the highest modulus of elasticity and maximum strength values were obtained at additive ratios of 0.10–0.15%. The highest tensile strength (55.31 MPa) and compressive strength (388.53 MPa) were obtained at ZnO contents of 0.10–0.15 wt%, while the maximum flexural strength reached 125.94 MPa at 0.15 wt% ZnO. In addition, the storage modulus increased from 1.469 × 10^9^ Pa for the control resin to 1.872 × 10^9^ Pa for the composite containing 0.15 wt% ZnO, indicating improved stiffness and thermomechanical stability. The stress–strain curves show that improvements in ductility and deformation capacity of the material are achieved at these additive ratios. The findings demonstrate that green-synthesized ZnO nanoparticles are an effective and sustainable additive material for improving the mechanical and thermal performance of DLP-based photopolymer dental resins.

## 1. Introduction

Three-dimensional (3D) printing technologies have revolutionized many industries by enabling the production of more complex geometries in shorter periods of time and with less material waste compared to traditional manufacturing methods. Among these technologies, photopolymerization-based methods such as stereolithography (SLA) are preferred, especially in applications requiring high resolution and surface quality [1]. In dentistry, photopolymer resins are widely used in the production of temporary restorations, surgical guides, and orthodontic plates [2]. However, these resins generally have a brittle structure and have disadvantages such as poor mechanical strength, limited biocompatibility and insufficient antimicrobial activity [3]. In recent years, various nanoparticle reinforcements have been used in the resin matrix to overcome these drawbacks. In particular, zinc oxide (ZnO) nanoparticles are a suitable candidate for many biomedical applications due to their high surface area, photoactivity under UV light, antimicrobial activity and electrical properties [4,5]. The special properties of nanoparticles which result from their high surface area and surface chemical activity show different biological responses than their larger material form. Recent research has shown that zinc nanoparticles produce bio-effects that are dose-dependent, as they exhibit antimicrobial properties at low concentrations and induce cytotoxic effects at higher concentrations. The dental and biomedical applications of ZnO nanoparticles in biomaterials require precise monitoring of their concentration and distribution throughout the polymer matrix [6].

The development of nano- and micro-composite materials suitable for 3D printing has been the focus of more research in recent years. The interest in these materials is based on the multi-scale interactions of nano- and micro-scale fillers with polymer matrices. The resulting composites show improved mechanical stability and enhanced durability and their structural integrity maintains its strength throughout time because of these interactions [7,8,9]. Comparative studies in the literature show that nano-scale fillers create better distribution within polymer matrices than micro-scale filler particles. The material achieves its structural performance through this property because it enables the material to reach higher mechanical toughness [10]. The addition of nanoparticles to the polymer matrix results in improved mechanical strength alongside enhanced properties which include surface hardness and heat resistance and light transmittance [11]. The process of distribution becomes harder because smaller nanoparticles have increased surface areas which cause their materials to clump together. The situation leads to decreased performance of the material according to its intended function [12]. The metal oxide nanoparticles display their maximum dispersion within the resin when researchers use high concentrations of ZnO nanoparticles. The presence of these nanoparticles in photopolymers causes three major alterations which include changes to the curing process, the light transmission properties, and the rate at which crosslinking occurs [13].

Traditional ZnO nanoparticle synthesis methods (such as sol–gel, precipitation, hydrothermal) often harm the environment due to the use of toxic chemicals and high energy requirements [14,15,16]. Therefore, in recent years, “green synthesis” methods have come to the forefront within the framework of sustainable production efforts [17,18,19,20]. Producing nanoparticles under environmentally friendly conditions using plant extracts, microorganisms, or natural polymers both provides energy efficiency and allows the production of biocompatible products.

Plant-based extracts are frequently preferred in ZnO NPs synthesis because they can act as both reducing and stabilizing agents [21]. Since the nanoparticles obtained in these methods have natural organic coatings on their surface, better interfacial interaction with the polymer matrix is achieved and the tendency for aggregation is reduced. In addition, studies have shown that phyto-mediated synthesized ZnO NPs show a superior antimicrobial effect compared to those synthesized by traditional methods [22]. In the literature, the integration of phyto-mediated synthesized ZnO NP-reinforced photopolymer resins into SLA-based production processes has been addressed in a limited number of cases.

The application of temporary dental restorations together with denture bases and occlusal splints and surgical guides needs materials that can provide both mechanical strength and dimensional stability and antimicrobial protection [23]. The moist, warm and microbiologically rich nature of the oral environment necessitates the use of bioactive, durable and biocompatible materials in these products. The use of ZnO NPs offers a great potential in this context, especially for treatment against caries-causing bacteria such as *Streptococcus mutans* [24]. In addition, properties such as curing depth, degree of polymerization, and dimensional stability of 3D printing resins are critically important for clinical success [25].

In recent years, it has been shown that some types of nanoparticles can not only provide structural reinforcement but also act as photoinitiators. In particular, semiconductor oxides (ZnO, TiO_2_, Fe_3_O_4_) and liquid metal gallium nanoparticles stand out as new-generation dopant materials that can initiate free radical polymerization in vinyl-based monomers [26,27,28]. When triggered by photons with energy equal to or greater than the band gap of the semiconductor, this initiates the separation of electron-hole pairs, triggering the mechanism of action for light-sensitive polymerization. Such an initiating capability for photosensitive polymerization can be further boosted by co-initiators such as tertiary amines or only slightly photoactive molecular surface modifications [29]. For example, non-photoactive molecules such as levulinic acid can be oxidized via photopath-generated holes when bound to the nanoparticle surface. At the same time, such surface modifications are also preferred to ensure more homogeneous distribution of nanoparticles in the polymer matrix [30]. Another important characteristic of nanoparticles is their size-dependent behavior. For example, it has been reported that ZnO quantum dots with a size of only 5 nm do not negatively affect the photopolymerization process initiated with camphorquinone or post-curing reactions but show segregation within the system according to the light pattern [31]. It has been stated that even some non-photoactive nanoparticles affect photopolymerization kinetics and some nanoparticles can increase the viscosity of the resin by absorbing, reflecting or scattering UV light. These effects, especially at high nanoparticle concentrations, necessitate the development of new strategies to improve or optimize the curing efficiency of the system. Studies have shown that low-power UV curing systems used by most entry-level 3D printers struggle to achieve complete conversion of the resin beyond its glass transition point [32]. That is, when the glass transition temperature (Tg) rises above the processing temperature, complete polymerization of the resin is prevented. Along with accelerated photokinetic reactions, mechanical stiffness provided by nanoparticle reinforcement can cause the glass transition point to shift to lower conversion levels, which can exacerbate this negative effect [33].

In this study, zinc oxide (ZnO) nanoparticles, synthesized using an environmentally friendly method with plant extract, were incorporated into polymethyl methacrylate (PMMA)-based photopolymer resin at different weight percentage ratios (wt% 0.05, 0.10, 0.15, 0.20, and 0.25), and samples were produced using an ASIGA Ultra brand digital light processing (DLP) 3D printer. For comparison purposes, pure (non-nano-modified) PMMA was also evaluated as a reference group. The obtained ZnO nanoparticles were characterized in terms of morphological, structural, and optical properties; subsequently, PMMA-ZnO composite samples with different doping ratios were comprehensively investigated using thermal analysis (TGA-DSC), dynamic mechanical analysis (DMA), Fourier transform infrared spectroscopy (FTIR), and bending, compression, and tensile mechanical tests. This work systematically investigates the structural and functional influence of phyto-mediated synthesized ZnO nanoparticles in photopolymer-based acrylic resins under DLP 3D printing conditions, providing a significant and pioneering contribution of both in the production of environmentally sustainable nanomaterials and in the domain of advanced dentistry.

### Objective of Present Study

A review of the current literature reveals that photopolymer resins reinforced with ZnO nanoparticles are mostly produced using conventional synthesis methods and are predominantly evaluated in SLA-based production systems. In contrast, the effects of ZnO nanoparticles obtained through an environmentally friendly plant extract-mediated synthesis method on the thermomechanical and mechanical behavior of PMMA-based dental photopolymers under DLP-based three-dimensional printing conditions have not been systematically addressed in the literature. Specifically, the optimum doping range, which defines the balance between curing efficiency, chain mobility, mechanical rigidity, and ductility depending on the nanoparticle doping ratio, has not been clearly established. The study of the effect of phyto-mediated synthesized ZnO nanoparticles on DLP-based PMMA photopolymer systems has the primary objective of determining the relative weight of mechanical tests and comprehensive thermomechanical analyses on a multiaxial basis. The study’s hypothesis states that phyto-mediated synthesized ZnO nanoparticles can be added to dental photopolymer resins in minimal amounts to improve their thermal and mechanical properties while their 3D printing processability and material strength remain intact.

Compared with earlier findings on chemically synthesized ZnO NPs, or classic PMMA systems, the present study has resolved the phyto-mediated synthesis method using *Dianthus chinensis* extract, then infused the latter into a DLP-printed dental photopolymer resin. Added to it was a systematic examination of thermomechanical behavior (DMA, TGA, DSC) along with mechanical properties (tensile, compressive and flexural analyses) to accommodate suitable concentrations of nanoparticle. This establishes a region of optimal balance for reinforcement topology (0.10–0.15 wt%) which will act as a guideline for further applications in the development of environmentally friendly nanocomposite resins meant for additive manufacture in dentistry.

## 2. Materials and Methods

A commercial dental photopolymer resin (Asiga DentaBASE PMMA, Asiga, Sydney, Australia) was used as the matrix material, while 0.05%, 0.10%, 0.15%, 0.20%, and 0.25% by weight of zinc oxide (ZnO) nanoparticles produced by the phyto-mediated synthesis process were mixed with the resin. According to the manufacturer’s safety data sheet (SDS), the material consists of methacrylate-based oligomers and photopolymerizable components rather than pure polymethyl methacrylate (PMMA). Such resins are commonly referred to as PMMA-like or methacrylate-based photopolymer systems used in additive manufacturing for dental applications.

### 2.1. Preparation of Aqueous Dianthus Chinensis Extract

In this study, *Dianthus chinensis* was utilized as a biological reducing and stabilizing agent. The samples were rigorously rinsed with deionized water to remove surface contaminants and subsequently dehydrated in a forced-air oven at 60 °C for 24 h. For the extraction process, 10 g of the dried petals were macerated in 100 mL of deionized water. The mixture underwent 2 h of aqueous extraction at 70 °C while being continuously stirred magnetically. After filtering the resultant suspension through a coarse porosity filter paper, the filtrate was kept for later use at 4 °C.

### 2.2. ZnO Nanoparticles Produced by Phyto-Mediated Synthesis Method

The biosynthesis of ZnO NPs was performed using zinc acetate dihydrate (Zn(CH_3_CO_2_)_2_·2H_2_O) as the metallic precursor and sodium hydroxide (NaOH) as the precipitating agent. Initially, zinc acetate (10 g) was completely dissolved in 22.8 mL of deionized water. In a typical synthesis procedure, 4 mL of the *D. chinensis* extract was introduced into the zinc acetate solution under continuous stirring. The reaction mixture was then graded with a 2 M NaOH solution, while stirring unceasingly at room temperature for 2 h. The resultant precipitate was filtered out and eliminated by sequential washing with deionized water (three times) and absolute ethanol (one time) to remove the leftover precursors and unreacted species. The purified nanoparticles were dried in an oven at 60 °C for 24 h. Finally, the samples underwent a programmed thermal treatment in a muffle furnace with a gradual ramp from 100 °C to 500 °C, followed by calcination at 500 °C for 4 h to achieve the desired crystalline phase. In this synthetic technique, the plant extract acts as the biological reducing and stabilizing agents, while zinc acetate serves as the precursor that provides Zn^2+^ ions for the further formation of nanoparticles.

### 2.3. Preparation of ZnO Nanoparticle-Reinforced Resin

Specified amounts of ZnO nanoparticles were gradually added to the liquid resin. To make the mixing highly uniform and eliminate the possibility of agglomerates, the mixtures were processed under controlled conditions with a high-shear-rate homogenizer (Ultra-Turrax, IKA, Staufen, Germany) at a stirring speed of approximately 5000 rpm for 2 min. The homogenization process continued until a visually stable and homogeneous suspension was obtained. The prepared nanocomposite resins were stored protected from ambient light and immediately subjected to 3D printing to prevent premature photopolymerization.

### 2.4. Specimen Fabrication via 3D Printing

**Figure 1:**
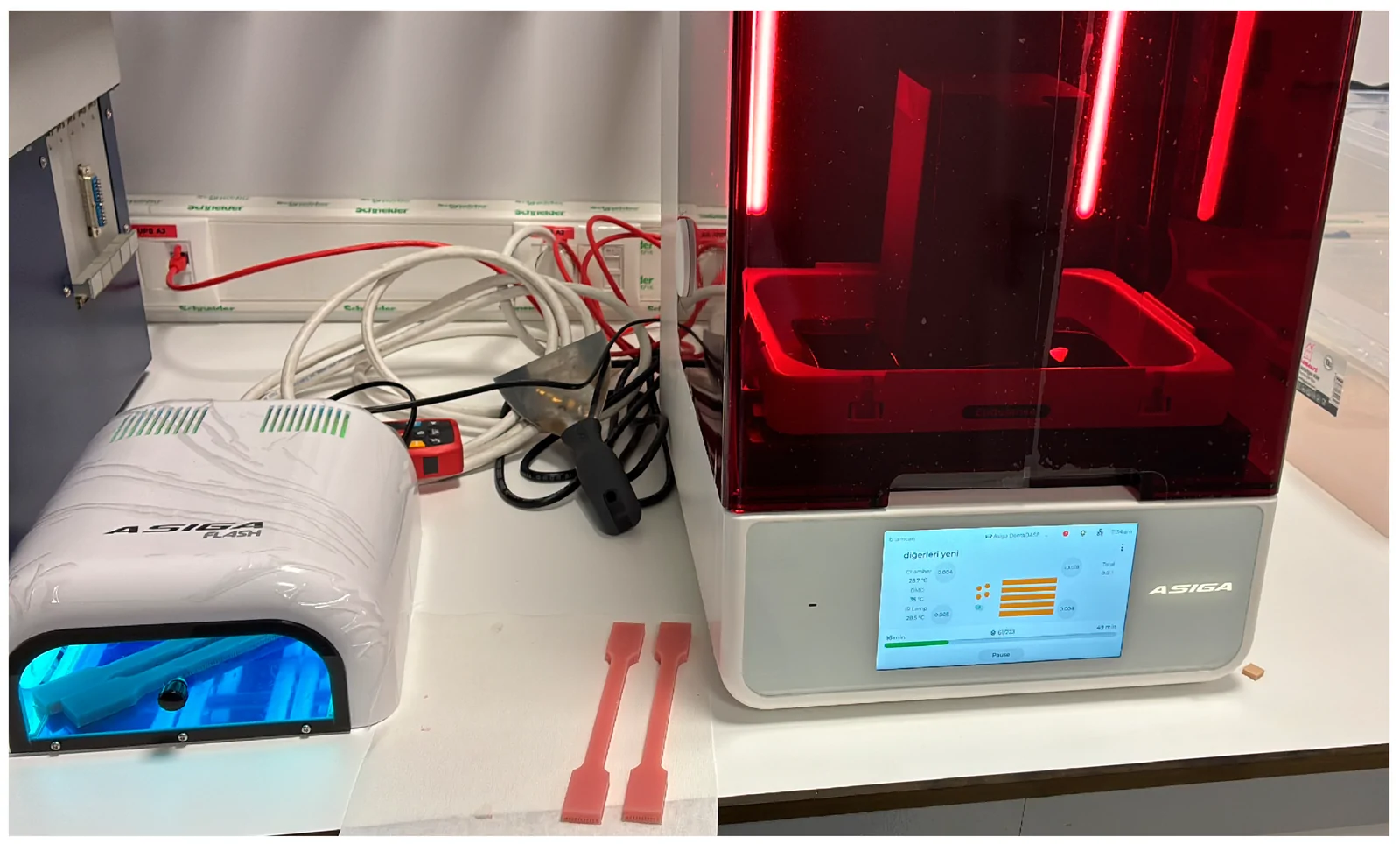
3D printer.

All samples were produced using a 3D printer (Asiga Ultra, Asiga, Sydney, Australia) with digital light processing (DLP)-based stereolithography (Figure 1). The printing process was performed with a layer thickness of 100 µm for all experimental groups; other parameters were kept constant according to the manufacturer’s recommendations. Sample geometries were modeled using computer-aided design (CAD) software (SolidWorks 2019, Dassault Systèmes, Vélizy-Villacoublay, France) and exported in STL format. Sample designs were created for three different tests as follows:

- For the tensile test: “dog-bone” shaped samples with a total length of 180 mm.
- For the flexural test: rectangular bars measuring 100 mm × 10 mm × 4 mm (support spacing: 80 mm).
- For the compressive test: cylindrical samples with a diameter of 10 mm and a height of 20 mm.

After printing, the samples were carefully removed from the platform, and uncured excess resin was removed. Then, post-curing was performed according to the manufacturer’s protocol to ensure consistent polymerization in each group. The specimens underwent cleaning procedures to eliminate remaining uncured resin after their printing process and they underwent post-curing treatment with a UV curing unit from Asiga Flash Cure Box (Sydney, Australia). The samples were exposed to post-curing for 60 min which included 30 min of treatment on each side according to the manufacturer’s specifications to achieve full photopolymer resin polymerization.

### 2.5. Characterization of ZnO Nanoparticles

The synthesized ZnO nanoparticles (NPs) underwent comprehensive characterization to determine their chemical, structural, and morphological properties using the following techniques:

- Chemical Composition and Functional Groups

The functional groups and surface chemistry of the synthesized ZnO NPs were investigated using FTIR spectroscopy (Nicolet 380 FTIR-ATR instrument, Thermo Scientific, Waltham, MA, USA). The spectra were recorded in the transmission mode across a spectral range of 4000 to 400 cm^−1^.

- Structural and Crystalline Properties

The structural characteristics of the synthesized ZnO nanoparticles were investigated using an X-Ray Diffraction (XRD) (PANalytical EMPYREAN, Almelo, The Netherlands). The diffraction patterns were recorded in a continuous scan mode over a degree range of 10° to 90°.

- Surface Morphology, Particle Size and Elemental Analysis

The surface morphology, agglomeration state, and particle sizes of the synthesized nanoparticles were examined using a Field Emission Scanning Electron Microscope (FESEM, Zeiss GeminiSEM 500, Carl Zeiss, Jena, Germany). The samples were coated with a thin layer of Gold prior to SEM analysis to increase conductivity and minimize charging effects. Image J software (version 1.54k) was used for the statistical analysis of the data obtained from the images. Diameter measurements were performed on a total of *n* = 100 randomly selected particles, and the average size and standard deviation values were calculated. The size distribution histogram was created using these numerical data. The elemental composition and surface purity of the synthesized ZnO NPs were determined using an Energy-Dispersive X-ray Spectrometer (EDS) integrated with the FESEM.

### 2.6. Thermal and Mechanical Characterization of Composites

The thermal and mechanical properties of the produced ZnO-reinforced composites were investigated using the following methods:

- Dynamic mechanical analysis (DMA)

Dynamic mechanical analysis (DMA) was employed to scrutinize the behaviors (mechanical and thermomechanical) of composites. The samples were held under the single cantilever bending mode, and the testing was done on a DMA 8000 machine when ramping the temperature to 200 °C at 3 °C/min with a 1 Hz frequency work alone. Measured parameters included storage modulus, loss modulus, and tan δ (damping factor). The results were analyzed to highlight the impact of adding ZnO on stiffness, energy dissipation, and glass transition behavior of the composites.

- Thermogravimetric Analysis (TGA) and Differential Scanning Calorimetry (DSC)

In order to study their thermal properties, samples were characterized by TGA and DSC. A Setaram-Labsys Evo instrument was also utilized in the analysis process under a nitrogen atmosphere, applying a heating rate of 10 °C/min. Additionally, analysis was carried out over a temperature range of about 800 °C from room temperature. The composites were subjected to TGA to determine their thermal degradation behavior and residual masses. Data from DSC were to be used to examine the glass transition temperature (Tg) and endothermic and exothermic peaks of phase transition. After changing each ZnO-dopant level, they sought to find out the effects of such of the dopant ratios in regard to their thermal stability.

- Fourier transform infrared spectroscopy (FTIR)

The FTIR analysis was applied to investigate the chemical bonding structure of ZnO nanoparticles and their interactions with the polymer matrix. Analyses were performed using a Thermo Scientific—Nicolet iS20 FTIR instrument (Waltham, MA, USA) in the range of 4000–400 cm^−1^ and with a resolution of 4 cm^−1^, via ATR probe. Both pure ZnO nanoparticles obtained by phyto-mediated synthesis and %ZnO-doped PMMA composite resins were analyzed by FTIR. This allowed for the comparative evaluation of the presence of Zn–O vibrational bands with characteristic PMMA bands such as ester, C=O, and C–H. The obtained spectra were interpreted in terms of the integration and chemical compatibility of the nanoparticles with the matrix.

**Figure 2:**
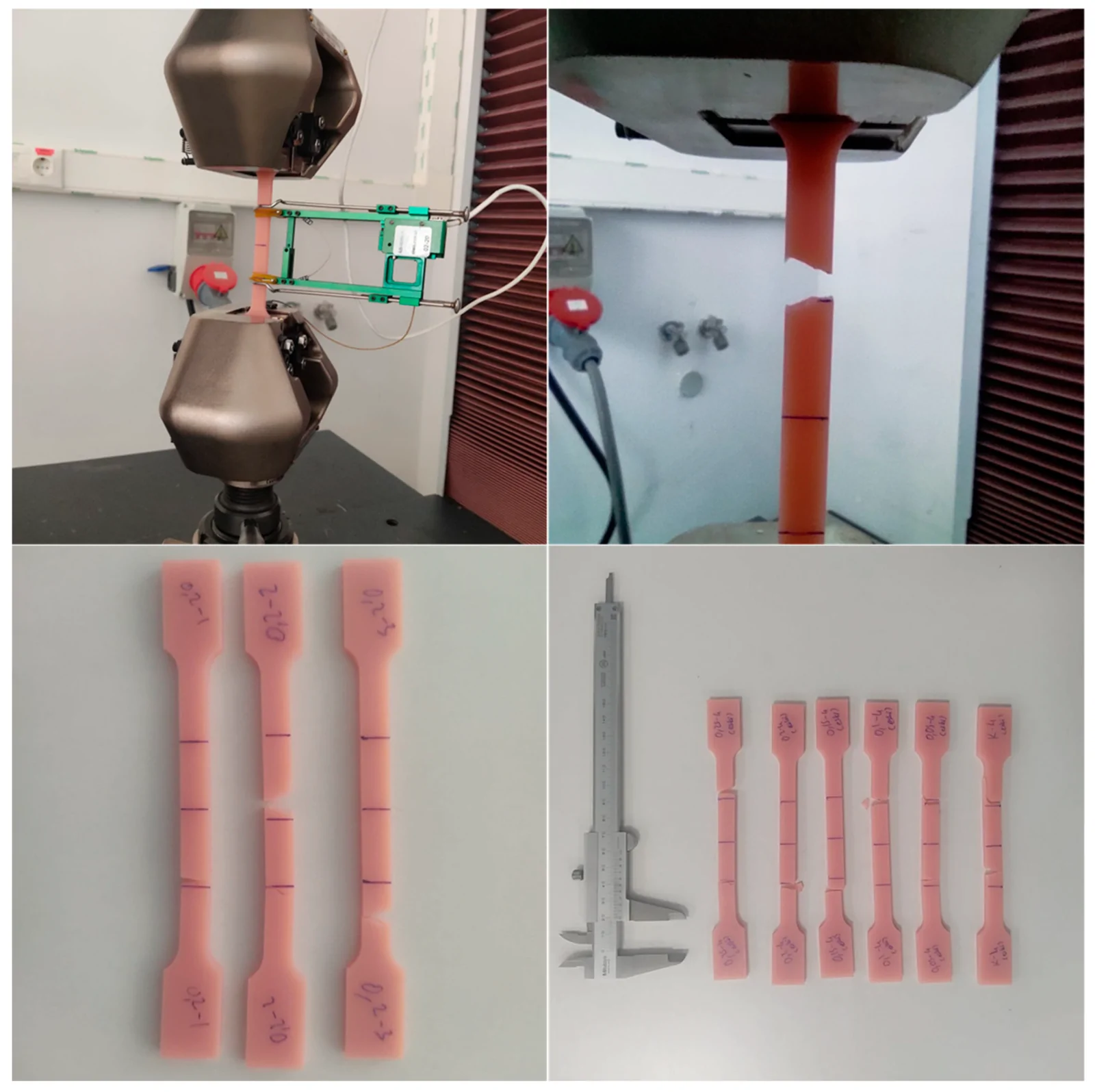
Tensile test setup and test specimens.

**Figure 3:**
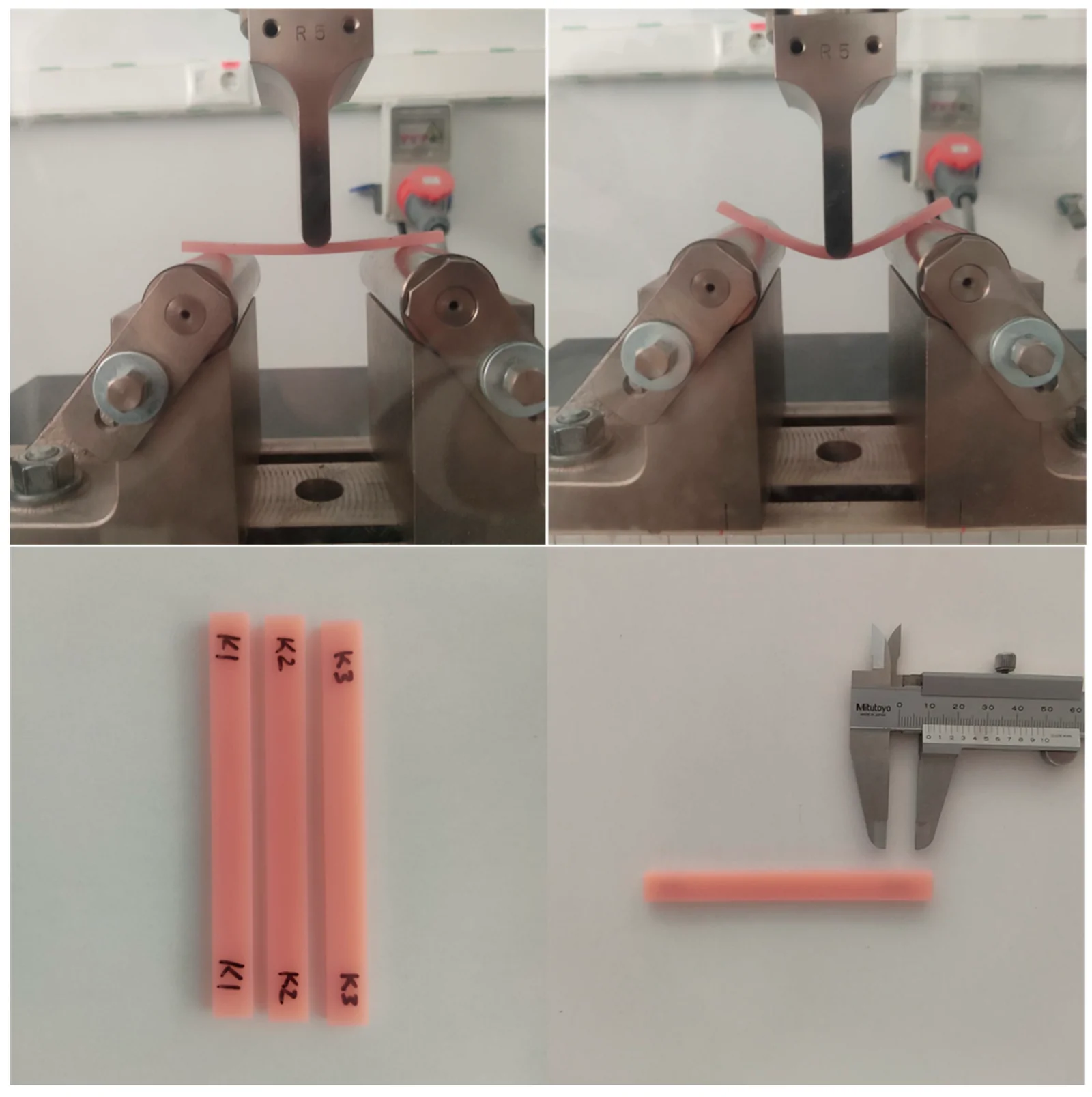
Flexure test setup and test specimens.

**Figure 4:**
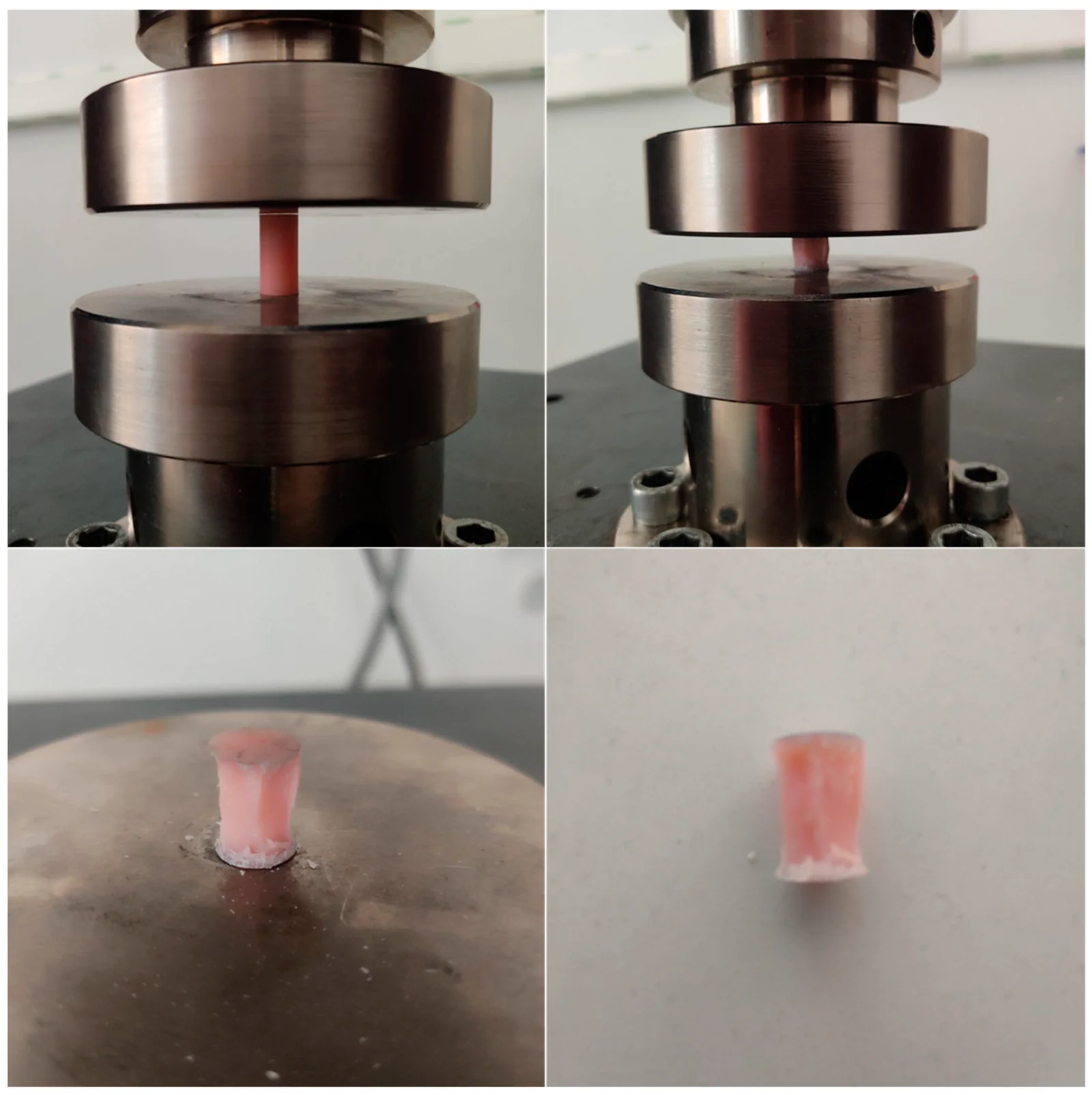
Compressive test setup and test specimens.

Mechanical tests:

- Tensile test: with dog-bone specimens (ASTM D638) (Figure 2).

- Flexural test: three-point bending method (ASTM D790, 80 mm between supports) (Figure 3).

- Compressive test: with cylindrical specimens (ASTM D695, Ø10 mm × 20 mm) (Figure 4) [34].

All tests were performed using a universal testing machine (Shimadzu AGS-X, Kyoto, Japan). Tensile strength, flexural strength, elastic modulus, and compressive strength were calculated from the obtained data. All tests were performed at room temperature (23 ± 2 °C), and at least three replicates (n = 3) were taken from each group for statistical validity.

These mechanical testing approaches are widely used for polymeric materials and provide results that can be discussed in relation to the requirements defined for dental base polymers in ISO 20795-1:2013 (Dentistry—Base polymers—Part 1) [35].

## 3. Results

### 3.1. Characterization Results of ZnO Nanoparticles

**Figure 5:**
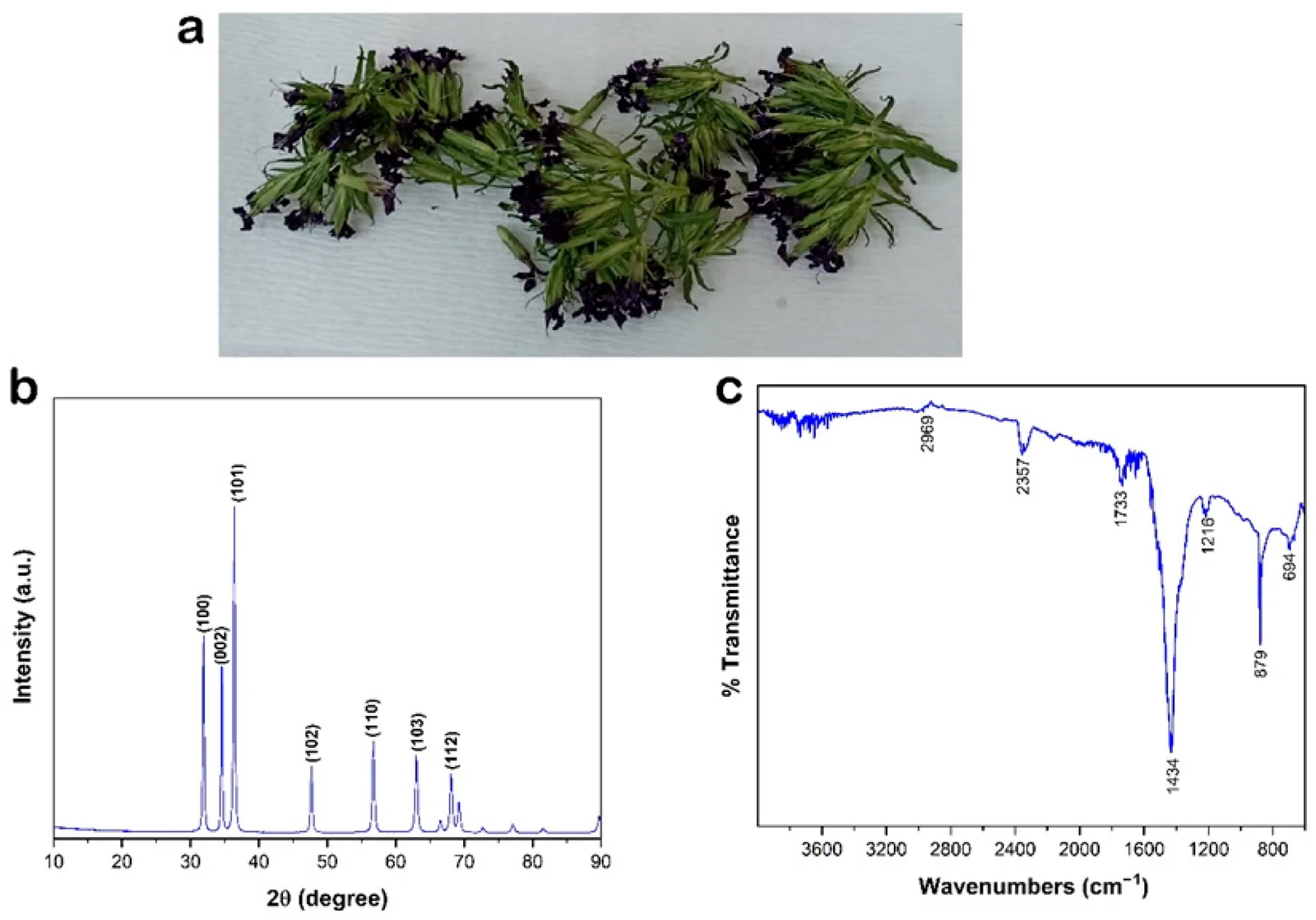
(**a**) Drying process of *D. chinensis* plant; (**b**) XRD pattern and (**c**) FTIR spectrum of the synthesized ZnO NPs.

XRD, FTIR, FESEM, and EDX analyses were employed to characterize the crystalline structure, surface functional groups, morphological features, and elemental composition of the synthesized ZnO NPs. The data obtained from these techniques confirms the successful phyto-mediated synthesis of high-purity zinc oxide nanostructures mediated by *D. chinensis* extract (Figure 5a).

#### 3.1.1. XRD Analysis

Figure 5b shows the XRD analysis results of ZnO NPs. The diffraction pattern was recorded over a two-theta range of 10° to 90°, revealing distinct peaks at two-theta positions of 31.89°, 34.57°, 36.41°, 47.69°, 56.73°, 62.97°, and 68.10°. These reflections correspond to the (100), (002), (101), (102), (110), (103), and (112) planes and are in agreement with the standard data reported in the Joint Committee on Powder Diffraction Standards (JCPDS) database (file no. 79-0208) [36]. The nanoparticles’ high crystallinity and lack of secondary impurity phases are indicated by the diffraction angles’ sharpness and high intensity [37].

#### 3.1.2. FTIR Analysis

The FTIR analysis provided a comprehensive understanding of the chemical interactions. As illustrated in Figure 5c, the structure of the ZnO NPs was confirmed by several characteristic absorption bands, including 2965 cm^−1^ (C–H stretching of aliphatic alkanes), 2357 cm^−1^ (asymmetric stretching of CO_2_ molecules or nitrile groups), and 1733 m^−1^ (C=O stretching of carbonyl groups in flavonoids and phenolic acids) [38]. The peaks at 1434 cm^−1^ and 1216 cm^−1^ are particularly significant, as they suggest the coordination of carboxylate and phenolic C–O groups to the zinc surface, forming a protective bio-organic shell. The peaks at 879 cm^−1^ and 694 cm^−1^ can be attributed to the vibrational bending of =C–H groups and O-H groups, respectively [39]. The weak absorption band exists in the low wavenumber range which extends from 500 to 600 cm^−1^ because of Zn–O stretching vibrations that prove ZnO nanoparticle formation. The additional bands identify organic functional groups that come from phytochemicals used in the phyto-mediated synthesis process.

#### 3.1.3. FESEM Analysis

**Figure 6:**
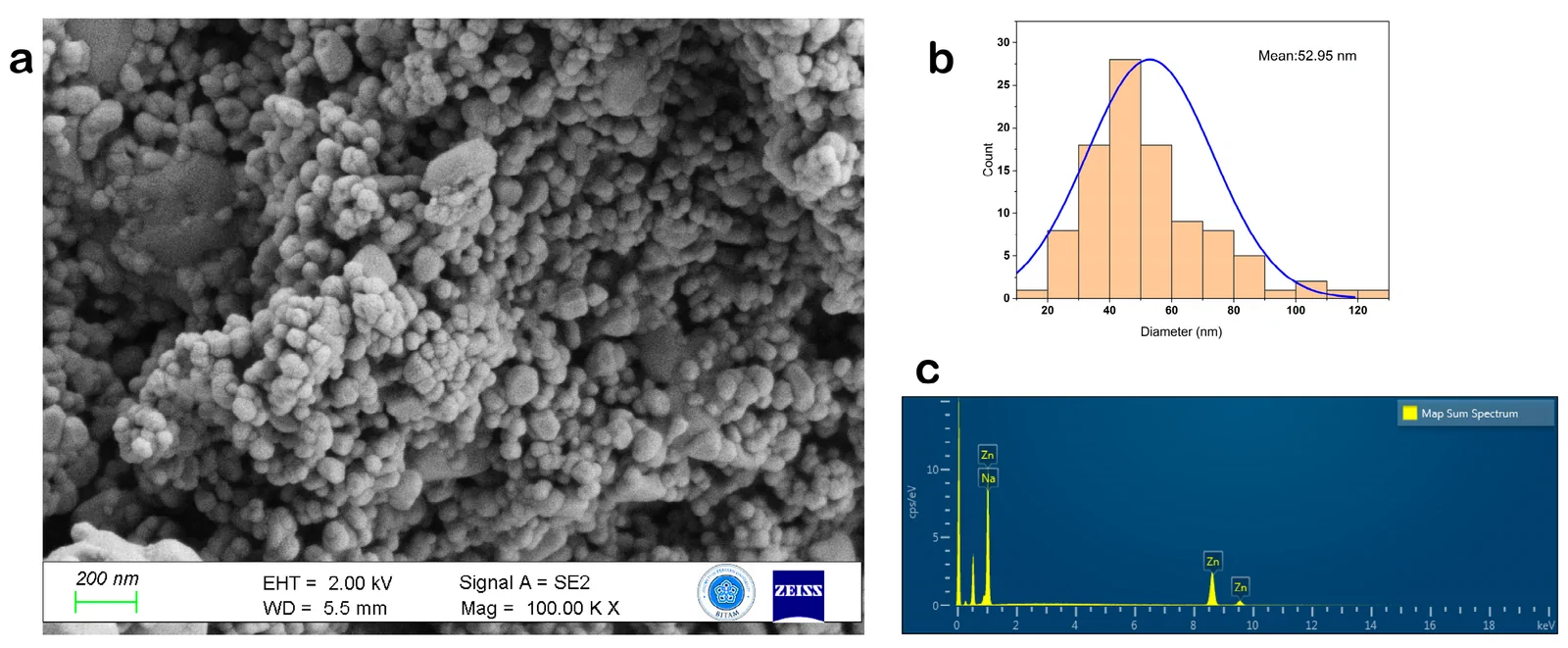
Characterization of ZnO NPs. (**a**) FESEM image revealing the surface morphology and agglomeration state of the nanoparticles, (**b**) particle size distribution histogram derived from FESEM images, (**c**) EDS spectrum confirming the elemental composition of nanoparticles.

FESEM images of the nanoparticles reveal that the particles generally exhibit a spherical morphology (Figure 6a). The investigations show that the nanoparticles tend to form agglomerated clusters held together by weak bonds rather than existing as individual particles. According to the statistical analysis results, the average diameter of the primary particles was determined to be 52.95 ± 20.29 nm (Figure 6b). It was observed that the particle size distribution was concentrated in the range of 35 nm to 55 nm and exhibited a narrow distribution (close to monodisperse).

#### 3.1.4. EDS Analysis

The elemental composition and purity of the ZnO nanoparticles synthesized using *Dianthus chinensis* extract were evaluated via EDS, as illustrated in Figure 6c. The nanoparticles exhibited characteristic peak lines at approximately 1.012 and 8.63 keV, which are attributed to Lα and Kα electronic transitions of Zn. EDS analysis confirmed the formation of ZnO NPs with weight ratios of 55.57% Zn and 26.73% O in the structure. The presence of carbon (C, 8.33%) observed in the spectrum indicates the presence of phytochemicals (polyphenols, proteins, or carbohydrates) in the plant extract that bind to the nanoparticle surface and provide stabilization. The sodium (Na, 9.37%) content may originate from precursors used for pH adjustment during synthesis or from the natural mineral content of the plant extract. The EDS spectrum shows a strong Zn peak which confirms the presence of ZnO nanoparticles. The sodium content in the sample derives from the NaOH which was used to control pH levels and the carbon content comes from phytochemical compounds that were extracted from plant materials during the phyto-mediated synthesis process.

### 3.2. Characterization Results of ZnO Nanocomposites

**Figure 7:**
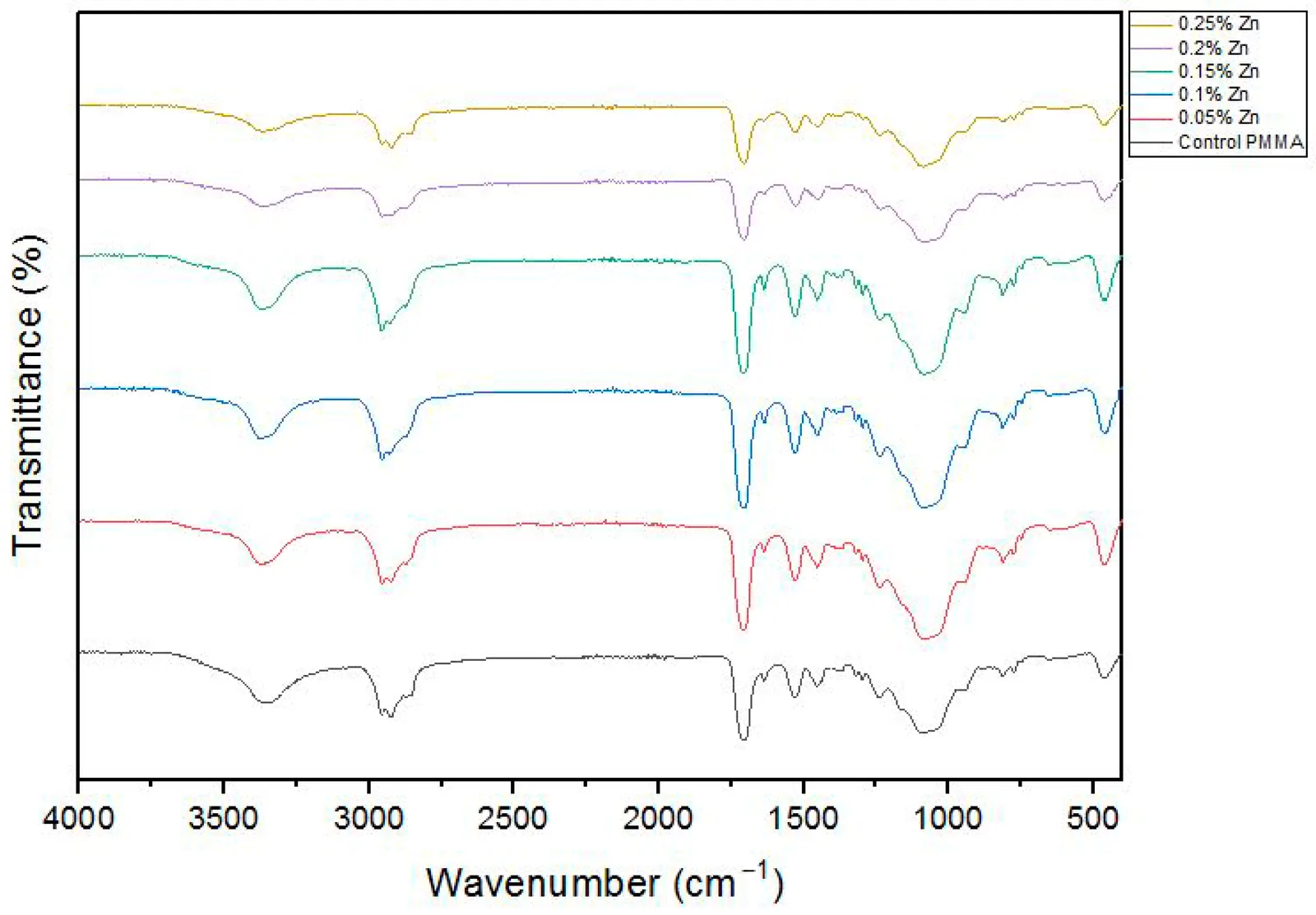
FTIR spectra of PMMA nanocomposites containing different ratios of ZnO.

Figure 7 shows the FTIR spectra of nanocomposite samples containing PMMA-based photopolymer resin (control group) and ZnO nanoparticles in varying concentrations from 0.05% to 0.25%. In all samples, characteristic absorption bands belonging to the PMMA structure were clearly observed. These include: a strong ester carbonyl (C=O) stretching vibration around 1720 cm^−1^, asymmetric and symmetric C–H stretching vibrations in the 2950–2850 cm^−1^ range, C–O–C stretching vibrations in the 1140–1190 cm^−1^ band, and a fingerprint region below 1000 cm^−1^. The doping of ZnO nanoparticles did not result in the formation of a new functional group; this indicates that no covalent bonding occurred between ZnO and the polymer matrix. In this special way, the nanoparticle and polymer system will possibly have some physical interactions, e.g., hydrogen bonds or van der Waals forces, due to the small wavelength shifts and intensity changes observed, particularly in the C=O and C–O regions. Furthermore, a weak and broad absorption around the 500–600 cm^−1^ band was observed only in ZnO-doped samples. This band corresponds to the stretching vibrations of the Zn–O bonds and confirms the successful incorporation of ZnO nanoparticles into the composite matrix. ZnO addition provided a physical modification that affected structural vibrations and altered the level of interaction in the PMMA matrix without leading to chemical bonding. The FTIR spectra show PMMA matrix absorption bands because of the low ZnO nanoparticle content which ranges from 0.05 to 0.25 weight percent. The different nanoparticle concentrations do not produce distinct spectral patterns because of this restriction, which results in only a minor Zn-O vibration band at low wavenumbers. The study used FTIR analysis to identify functional groups because the ZnO nanoparticle content was too low for detection at 0.05–0.25 wt%. The researchers did not use FTIR spectroscopy to measure the different amounts of nanoparticles in their samples.

**Figure 8:**
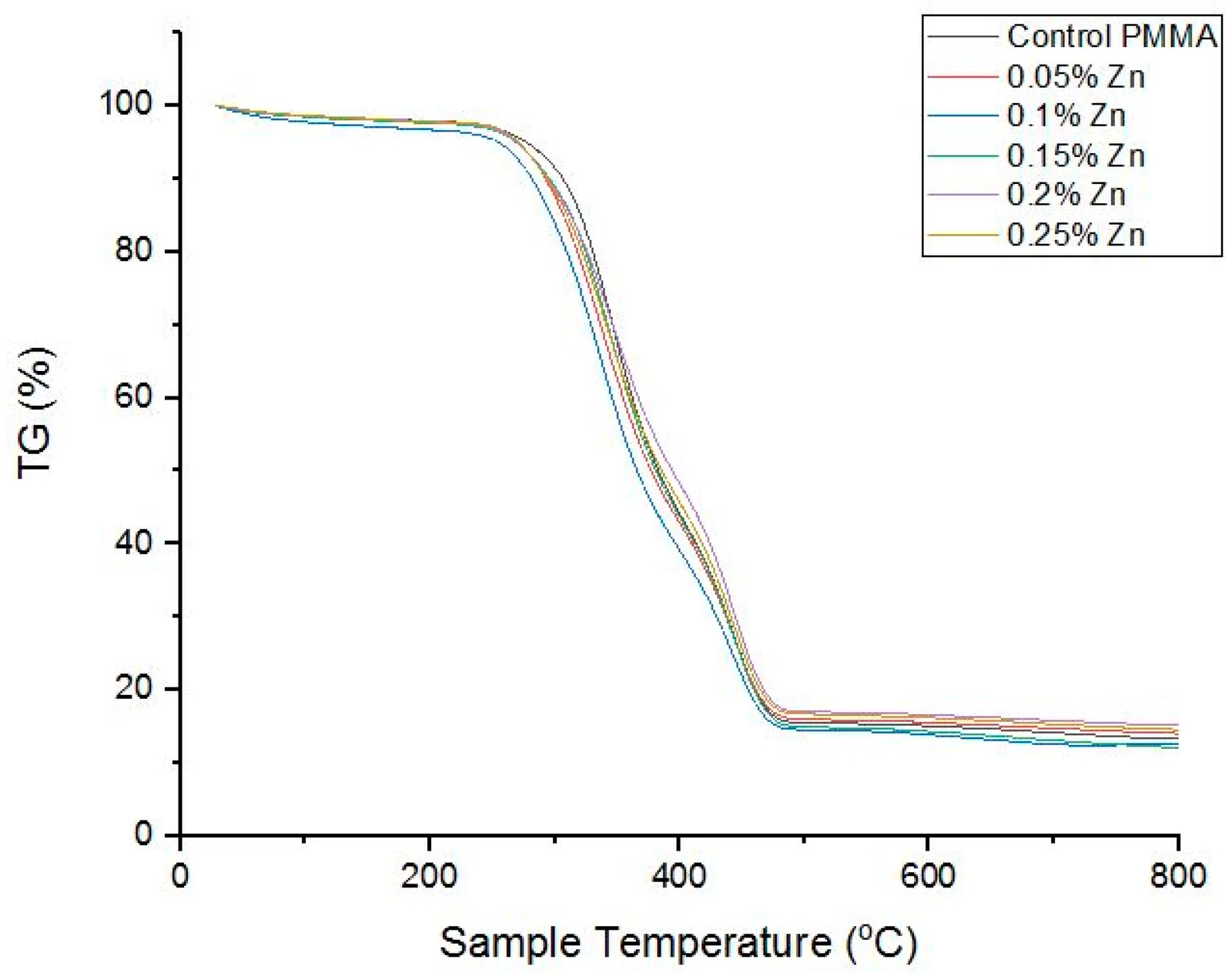
TGA curves of ZnO-reinforced PMMA composites (mass loss in %).

**Figure 9:**
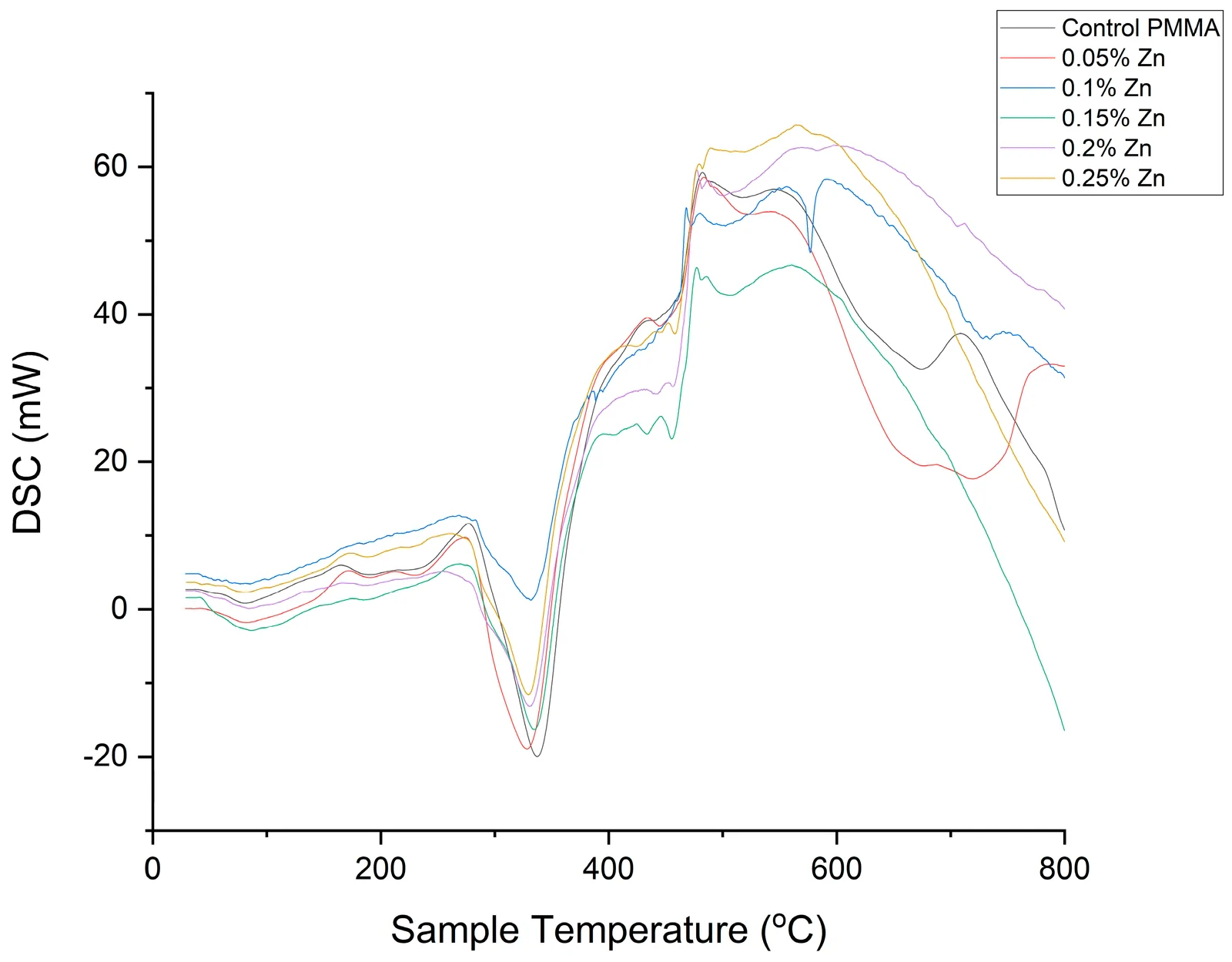
DSC curves (heat flux in mW) of ZnO-reinforced PMMA composites.

Figure 8 and Figure 9 show the TGA and DSC analysis results of nanocomposites containing ZnO nanoparticles and a control group PMMA-based photopolymer resin. A similar thermal degradation trend was observed in all samples, with degradation occurring approximately in the temperature range of 350–420 °C. The rapid mass loss occurring in this range represents the main thermal degradation region of the PMMA structure. In samples containing ZnO additive, especially at 0.15% and higher additive levels, a partial increase in thermal stability and a shift in mass loss temperature to higher values were observed.

According to DSC analysis results, endothermic reactions were observed in all samples at around 400–430 °C (Figure 9). This transformation is a result of heat absorption during PMMA degradation: several small shifts in terms of peak positions as well as intensities can be observed for the endothermic transitions that have been encountered in the four cases that included ZnO in the samples. In particular, the heat flux values were higher in samples with 0.2% and 0.25% ZnO additive, which is thought to indicate a more intense particle–matrix interaction.

**Figure 10:**
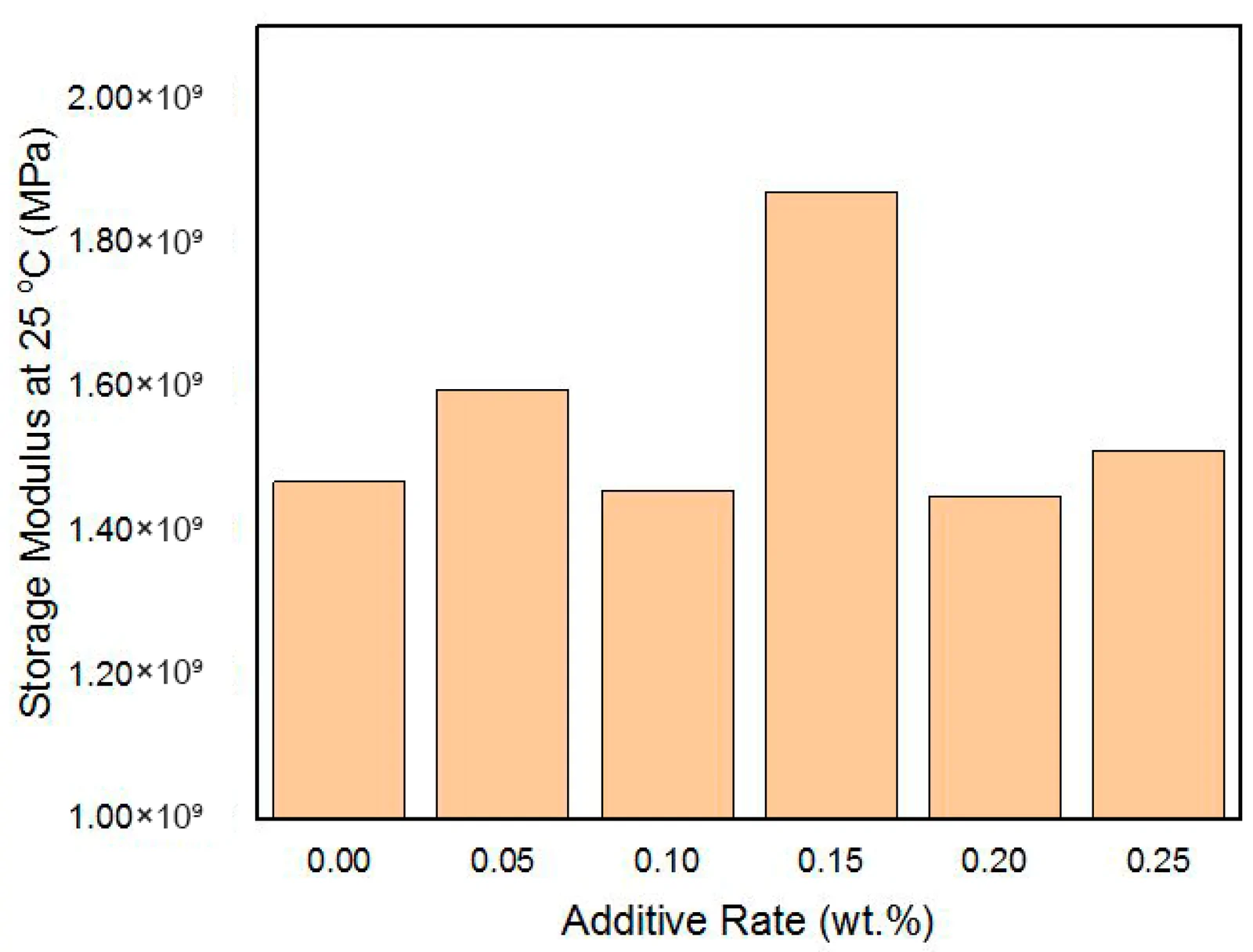
Storage modulus values of PMMA nanocomposites obtained with different ZnO doping ratios at 25 °C.

Figure 10 shows the storage modulus values of PMMA composites with different ZnO nanoparticle doping ratios measured at 25 °C. Storage modulus is an important parameter for determining the elastic energy storage capability of the polymer matrix. It was approximately 1.469 ×10^9^ MPa in the control group. The highest value reached in 0.15% ZnO-doped compounds was 1.872 ×10^9^ MPa. This shows that, up to a certain doping ratio, ZnO nanoparticles disperse more effectively within the matrix, increasing the stiffness of the structure. However, structural degradation due to particle agglomeration caused a decrease in the modulus again in samples exceeding the 0.15% doping ratio.

**Figure 11:**
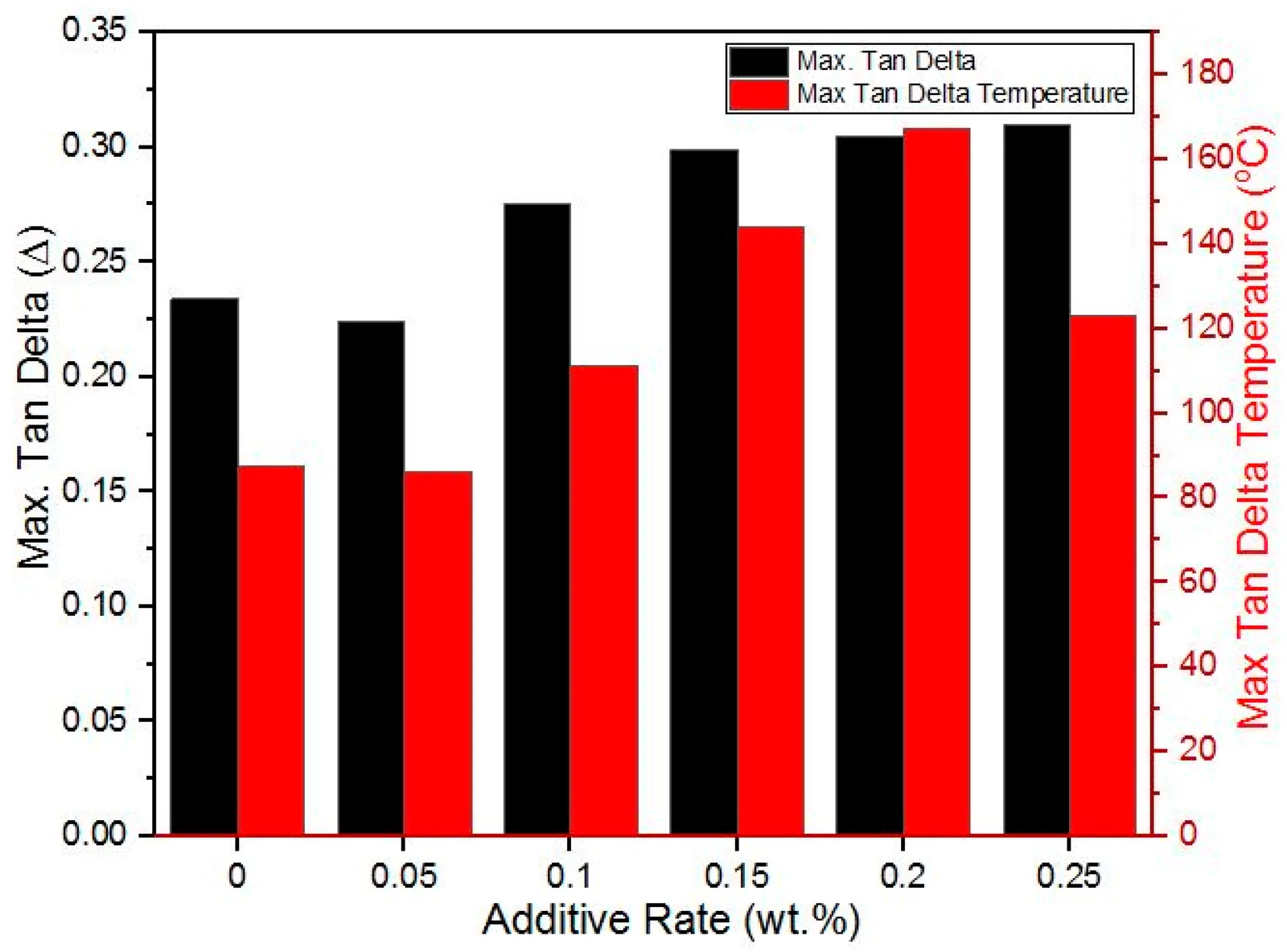
Comparison of maximum tan δ values and their corresponding glass transition temperatures (Tg) according to ZnO-dopant ratio.

The maximum *tan* δ (damping factor) values obtained for each additive ratio and the corresponding glass transition temperatures (Tg) are shown in Figure 11. *Tan* δ represents the internal damping capacity of the material, while the Tg value determines the temperature at which the polymer transitions from the amorphous phase to the rubbery phase. When the results are examined, it is seen that the *tan* δ values generally increase with the addition of ZnO, and the highest value is obtained in the sample with 0.2% ZnO additive. This is due to the nanoparticles interacting with the matrix, providing greater energy dissipation. When the Tg values are examined, it is seen that Tg increases significantly as the additive ratio increases, and the highest Tg value is also found in the sample with 0.2% ZnO additive (~170 °C). This indicates that the polymer chain mobility of the nanoparticles is restricted, and the thermal resistance is increased.

The observed increase in glass transition temperature (Tg) upon addition of ZnO nanoparticles indicates that the nanoparticles have a limiting effect on polymer chain mobility (Figure 9 and Figure 11). At low addition rates (≤0.15 wt.%), ZnO nanoparticles were more homogeneously distributed within the matrix, contributing to a more rigid structure by increasing polymer–particle interface interactions. In contrast, at higher addition rates (≥0.20 wt.%), the increased tendency of nanoparticles to scatter light and agglomerate negatively affected curing homogeneity and led to irregularities in chain mobility. This behavior is consistent with the observed decrease in mechanical performance despite the increase in Tg values.

### 3.3. Mechanical Properties of Nano Composites

#### 3.3.1. Elasticity Modulus (Young’s Modulus)

**Figure 12:**
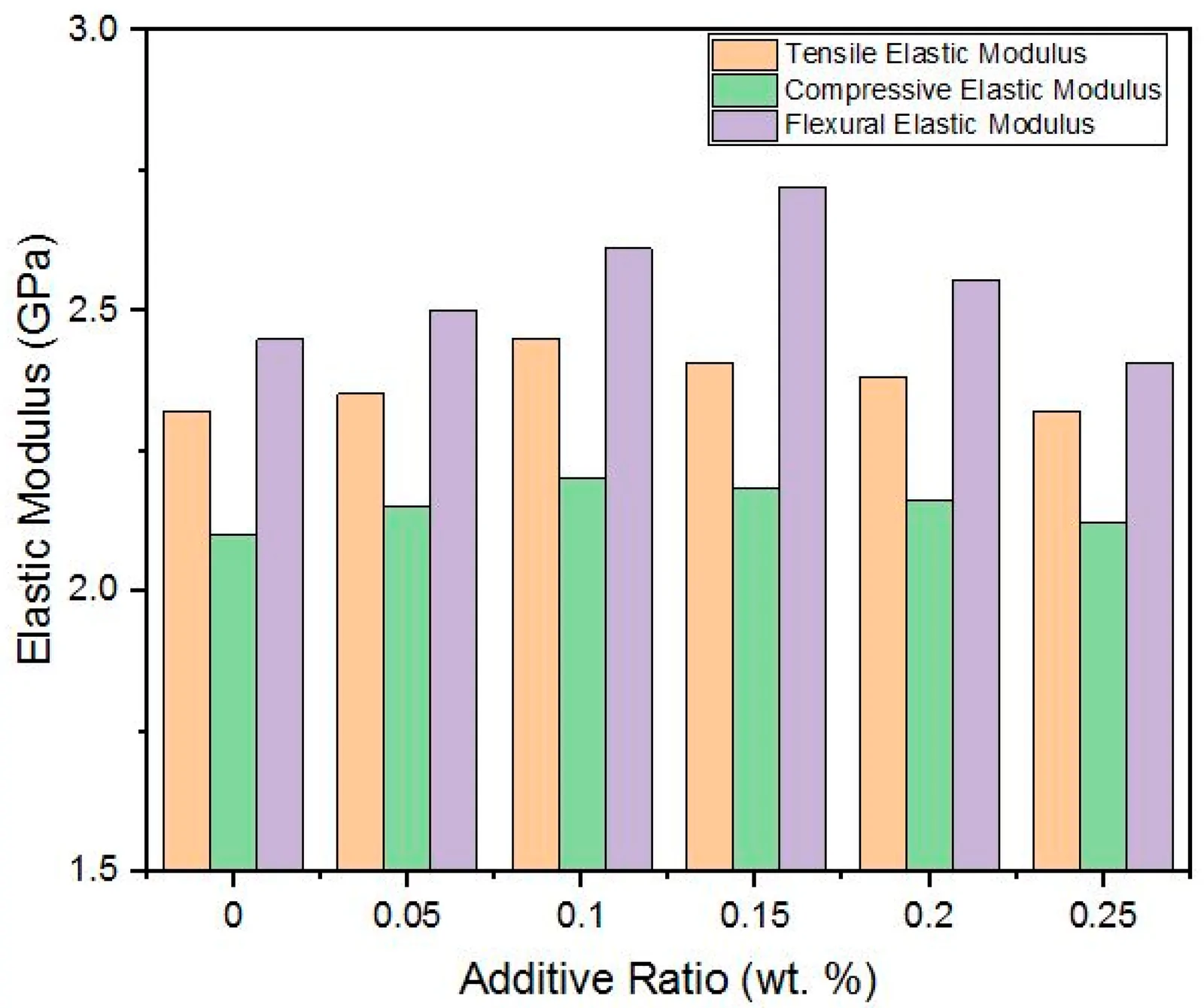
Tensile, compressive and flexural modulus of elasticity values (GPa) of PMMA composites produced with different ZnO addition ratios.

The tensile, compressive, and flexural modulus of elasticity values of PMMA composites produced with different ZnO nanoparticle addition ratios are given comparatively in Figure 12. In the presence of all types of tests, the modulus of elasticity changed significantly due to ZnO addition. The tensile modulus of elasticity displayed a general increase as the additive ratio increased. The value, which was 2.321 GPa in the control group, reached a maximum of 2.451 GPa with 0.1% ZnO additive. It was observed at 2.406 GPa with 0.15% additive, and this value decreased again to 2.312 GPa with 0.25% additive. Similarly, the compressive elasticity modulus reached a maximum value of 2.202 GPa with 0.1% ZnO additive, indicating that the ZnO additive increased the rigidity of the structure. The flexural modulus of elasticity is the parameter most sensitive to the additive ratio. This property, which was 2.45 GPa for the control group, attained its highest value of 2.722 GPa when the ZnO amount increased by 0.15%. This means that the ZnO particles do provide effective loading transfer, especially in flexural strain.

ZnO addition between 0.10 and 0.15% had the most positive effect on the elastic behavior of the composite. At higher addition rates (especially 0.25%), decreases in the modulus of elasticity were observed, which is likely related to nanoparticle agglomeration and matrix degradation. The subsequent decrease in elasticity modulus at 0.25 percent ZnO is in line with the literature observation that excessive reinforcement results in clumping and matrix degradation [40].

#### 3.3.2. Maximum Strength

**Figure 13:**
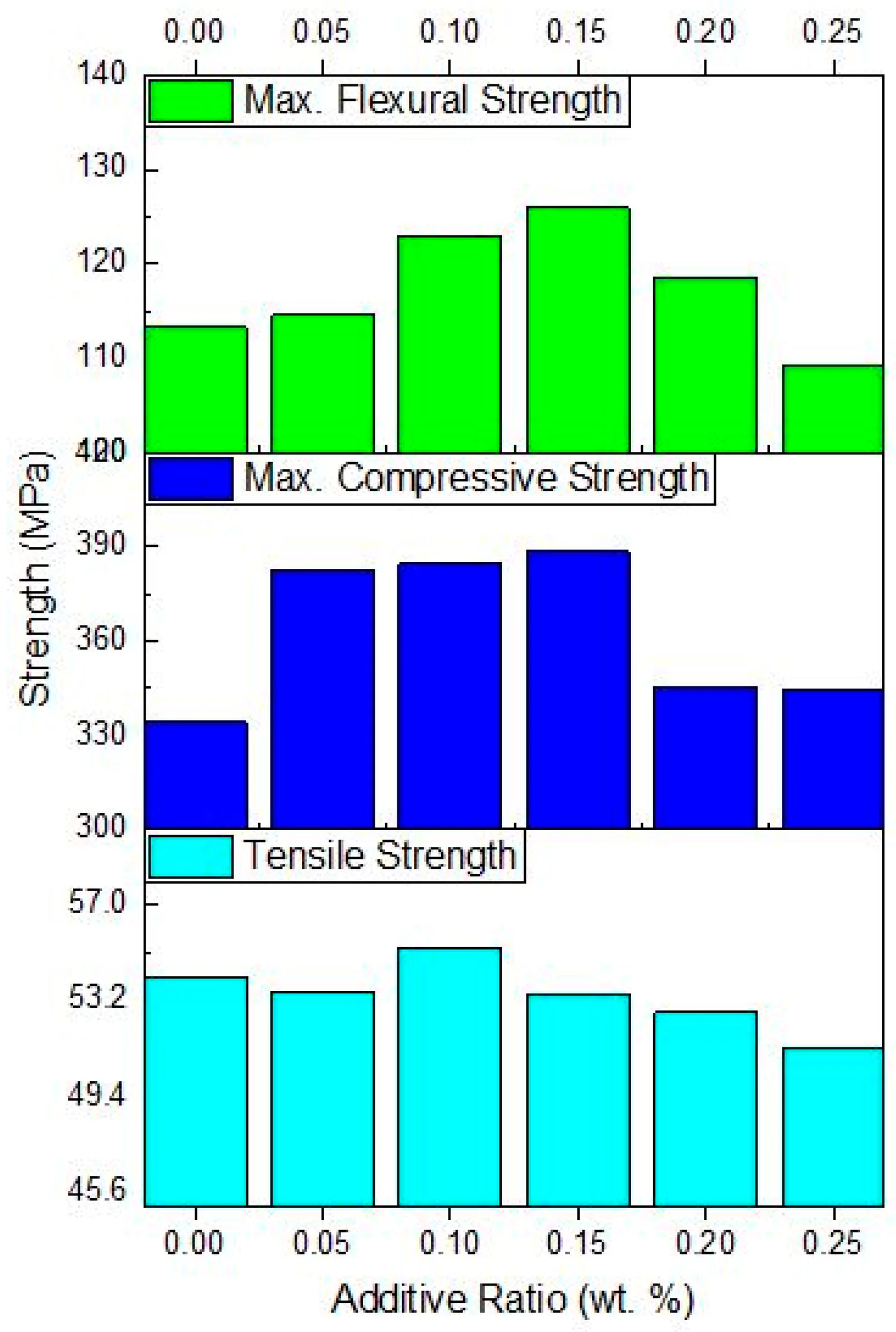
Maximum tensile, compressive, and flexural strength values obtained in PMMA nanocomposites according to ZnO addition ratios.

The maximum stress values obtained from tensile, compressive, and flexural tests of PMMA nanocomposites produced with different ZnO doping ratios are compared in Figure 13. Tensile strength showed slight fluctuations depending on the additive ratio. The value, measured as 54.11 MPa in the control group, reached a maximum of 55.31 MPa with a 0.1% ZnO additive ratio. However, one of the detractive effects of increasing amounts of additives was the decrease in strength, and thus tensile strength was reduced to 51.28 MPa on average in samples containing 0.25% ZnO additive. Conversely, practically no compressive strength in the control group gave rise to a significant increase in the compressive strength as ZnO was added. The maximum compressive strength in the control group was recorded to be 334.37 MPa, which rose to 388.53 MPa at a point of 0.15% ZnO incorporation. Similar increases were observed with 0.1% and 0.05% additions. Bending strength stands out as one of the most additive-sensitive parameters. This value, measured at 113.29 MPa in the control sample, reached its highest level at 125.94 MPa with a 0.15% ZnO additive rate. In general, the additive range of 0.10–0.15% has been determined as the most suitable range for improving the mechanical strength of the composite. A decrease in strength was observed at higher additive ratios.

#### 3.3.3. Stress–Strain Behavior

**Figure 14:**
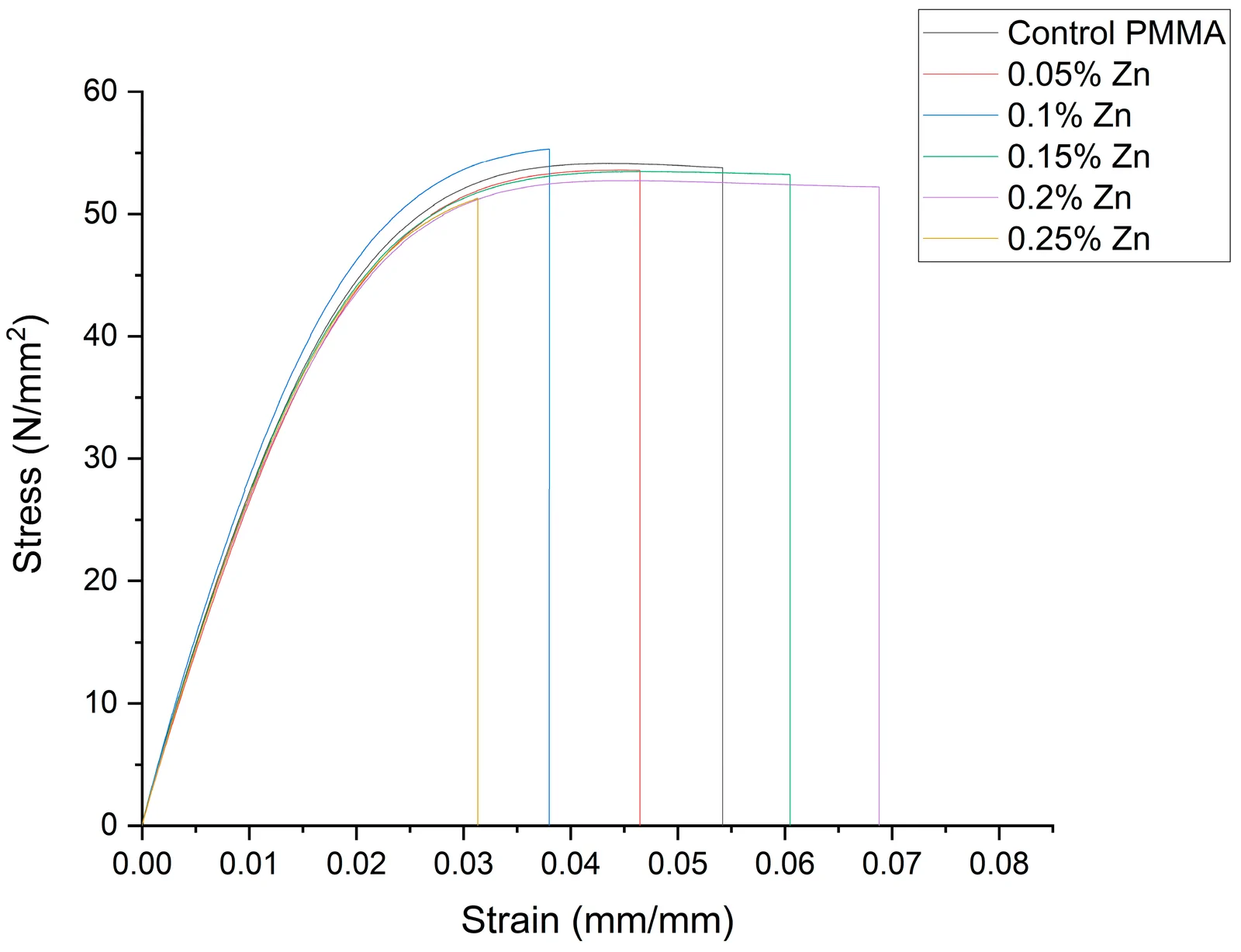
Stress–strain curves obtained from tensile testing of PMMA nanocomposites with different ZnO doping ratios.

The stress–strain curves obtained during tensile tests are presented comparatively in Figure 14. In all samples, the initial slope represents elastic behavior; the increase in slope in this region is consistent with the increase in the modulus of elasticity depending on the additive ratio. The highest slope was observed in the sample with 0.10% ZnO additive, indicating the formation of a more rigid structure. When fracture elongations were evaluated, samples with 0.15% and 0.20% ZnO additives achieved the highest deformation capacity (approximately 0.06 mm/mm). This indicates that ductility increases at these additive ratios and the material can absorb more deformation before fracture. The sample with 0.25% additive fractured earlier, showing the lowest strain value (~0.03 mm/mm).

**Figure 15:**
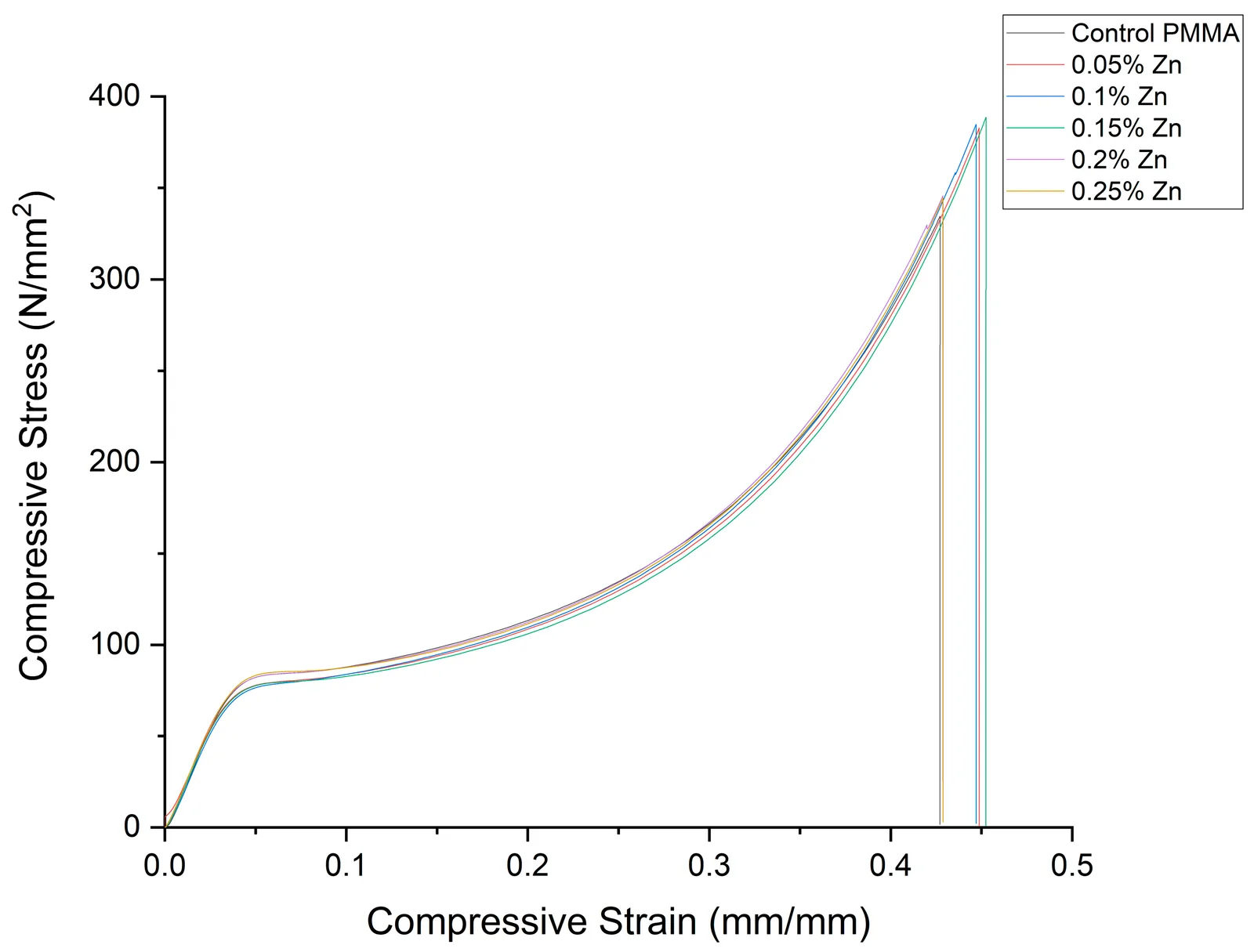
Stress–strain curves obtained in compressive tests of PMMA nanocomposites according to ZnO doping ratios.

Stress–strain curves obtained from compression tests are given in Figure 15. In all samples, a yield-like plateau region forms after the elastic region, followed by a continuously increasing non-linear rise. Samples with 0.10 and 0.15 wt.% ZnO additive showed deformation capacities reaching approximately 0.45 mm/mm. Both strength and deformation capacity increased in these samples, indicating that the ZnO additive had a supportive effect on mechanical absorption. In the control sample, the maximum compressive strength was measured as 334.37 MPa, and the yield strength as 85.25 MPa. In contrast, in the sample with 0.15% ZnO additive, these values reached 388.53 MPa and 87.12 MPa, respectively, showing the highest compressive strength. With 0.10% additive, the yield strength increased to its highest level, 90.01 MPa. However, in the sample with 0.25% ZnO additive, earlier fracture (approximately 0.38 mm/mm) and lower strength (344.38 MPa) were observed.

**Figure 16:**
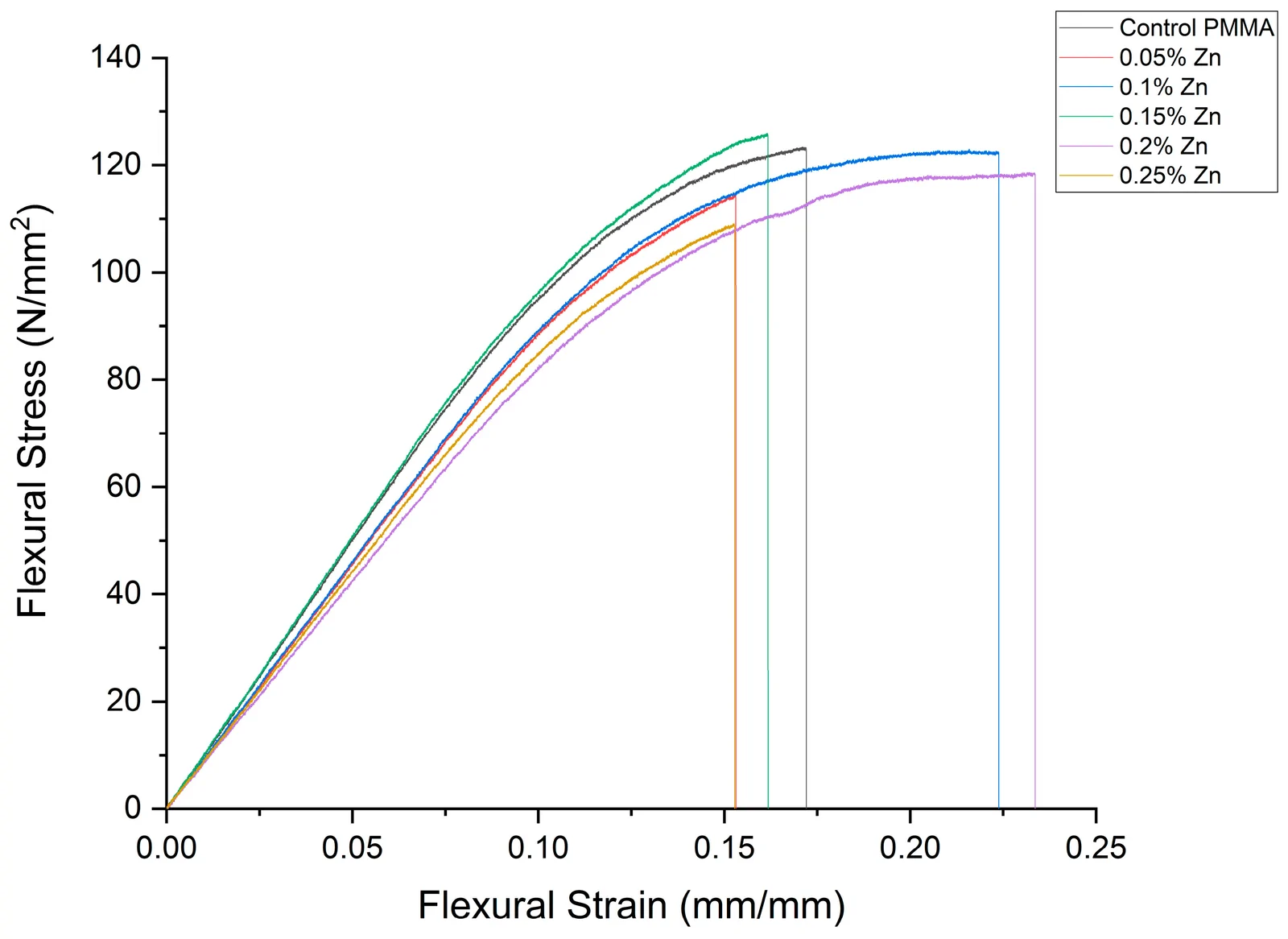
Stress–strain curves obtained from bending tests of PMMA nanocomposites produced with different ZnO doping ratios.

Stress–strain curves obtained from bending tests of composites are given in Figure 16. In all samples, a region of plastic deformation was observed following elastic behavior, and then fracture occurred. The specimen with the ZNO additive of 0.15% and 0.10% had achieved higher stress values with a ramped curve going through a steeper slope. The group of specimens with ZrO2 additives was found to have variable properties. The highest bending strength values were obtained by 0.15%-ZnO. The values are above 125.94 MPa, imputing an increase of about 11% compared to the control-based charge. This slight increase in the charging of charge suggests that ZnO nanoparticles provide better inter-chain charge transfer between polymer chains. ZnO addition positively affected the flexural strength of the PMMA matrix up to a certain ratio, but excessive addition weakened the mechanical performance. This behavior indicates that the ZnO nanoparticles facilitate a better transfer of charge between polymer chains, thereby increasing overall structural integrity. These findings are strongly in line with the findings of Khlifi et al. (2023), which claimed that, at low concentrations, the density of polymer chains occurring in the immediate vicinity of nanoparticles results in enhanced mechanical properties [41].

The fact that the ZnO additive provides maximum mechanical performance in the range of 0.10–0.15% indicates that nanoparticles can effectively perform charge transfer at these additive levels (Figure 12 and Figure 13). Above these ratios, the tendency of nanoparticles to agglomerate increases, and these clustered structures form stress concentration zones, acting as crack-initiating defects. At high additive ratios, a decrease occurs in the modulus of elasticity and maximum strength values (Figure 14, Figure 15 and Figure 16). This reveals that determining the optimum additive ratio is critically important in nanofilled photopolymer systems, and that mechanical performance depends not only on the amount of filler but also on the dispersion quality [42,43].

The mechanical improvements found in this study match earlier findings about nanoparticle-reinforced polymer systems which scientists studied for their biomedical and dental applications. Recent studies have reported that the incorporation of ZnO-based nanofillers into polymer matrices can enhance mechanical strength, stiffness, and functional performance due to improved stress transfer at the polymer–nanoparticle interface. Similar trends have been reported for nanoparticle-modified photopolymer systems, which show that low nanoparticle concentrations lead to better mechanical performance, whereas high filler contents result in particle agglomeration, which decreases performance. The optimum performance observed in this study at ZnO concentrations of 0.10–0.15 wt% is therefore in good agreement with results reported in recent studies on ZnO-reinforced photopolymer and biomedical composite materials [44,45,46].

The mechanical and thermal performance considerations, one has to be concerned with the possible biological impacts of ZnO nanoparticles for use in dentistry, given some events from previous reports showing that exposure to ZnO nanoparticles brings about biological responses which are dose-dependent. At long intervals, ZnO nanoparticles are generally thought to cause or are even reported to cause some unspecific antibacterialization stimulation against an oral pathogen like Streptococcus mutans at a low concentration. However, at higher concentrations, these nanoparticles may cause cytotoxic effects accompanied by an increase in the generation of Reactive Oxygen Species (ROS) and the release of Zn^2+^ ions. The present research restricted the usage of ZnO nanoparticles to a low concentration range which extends from 0.05 percent to 0.25 percent weight. Multiple studies demonstrate that the addition of ZnO nanoparticles below 1 percent weight induces no major cytotoxic effects while delivering functional benefits which include antimicrobial effectiveness and improved mechanical strength. The research used nanoparticle concentrations which dental material applications consider as safe. Further biological assessments which include in vitro cytotoxicity and antimicrobial testing should happen to support upcoming research.

The maximum stress values obtained for each contribution ratio are presented comparatively in Table 1. This table numerically summarizes how mechanical performance changes depending on the contribution ratio.

The improvement in mechanical properties observed in the present study is consistent with previous reports on nanoparticle-reinforced dental polymers. Several studies have shown that the incorporation of metal oxide nanoparticles such as ZnO into PMMA-based matrices can significantly enhance stiffness and strength due to improved stress transfer between the polymer chains and the dispersed nanoparticles [1,41].

## 4. Conclusions

In this study, the structural, thermal, and mechanical behaviors of nanocomposites formed by incorporating ZnO nanoparticles, obtained by phyto-mediated synthesis using *Dianthus chinensis* plant extract, into three-dimensional printable PMMA-based photopolymer resins at various ratios (0.05–0.25% wt), were comprehensively evaluated. FTIR analyses revealed that ZnO nanoparticles integrate with the polymer matrix through physical interaction rather than chemical bonding. Thermal analyses (TGA and DSC) showed that ZnO addition, particularly at a 0.15% addition rate, increased thermal stability and led to an increase in the glass transition temperature (Tg). This statement shows particles that enhance thermal performance by limiting the mobility of polymer chains. On the basis of observations, with the addition of ZnO, DMA results revealed great increases in storage modulus and Tg values, thereby giving credit to the stiffness of the structure of the particles.

Structural and mechanical testing performed characteristic tensile, compressive and flexural tests showing hit and miss responses depending on the addition ratio with the highest strength and elastic stiffness obtained at the 0.10–0.15% addition ratio. Stress–strain curves revealed that ductility and energy absorption capacity improved at these addition ratios. The reduction in mechanical properties is seen to be induced due to agglomeration effects when the amount of atoms was 0.20–0.60% and above.

This study has demonstrated that ZnO nanoparticle doping is an effective way to improve the thermal and mechanical performances of photopolymer dental materials produced by an environmentally friendly method, as well as strongly contributing to the field in biomaterial exploration aimed at DLP-based 3D printing.

The outcome of this study showed that ZnO-NPs prepared by a plant extract-based phyto-mediated synthesis method improve the thermal and mechanical properties and yield significant environmental claims. Prescribed additive content limits reported (0.10–0.15 wt%) can offer an apposite designing framework to achieve a performance balance amongst stiffness, strength, and ductility in 3DLP-based occlusive-dental photopolymer systems. This study therefore provides useful insights toward the sustainable development of next-generation dental photopolymer materials by introducing functional performance improvements alongside a sustainable production approach.

Some limitations of this study should be noted. The study used a limited number of mechanical testing specimens because researchers did not conduct detailed statistical analysis. Future studies should include larger sample sizes and biological evaluations to create a complete assessment of the nanocomposite materials.

## Figures and Tables

**Table 1:** Maximum tensile, compressive, and flexural stress values of PMMA composites with different ZnO content ratios.

Specimen	Tensile Elastic Modulus (GPa)	Compres-Sive Elastic Modulus (GPa)	Flexural Elastic Modulus (GPa)	Tensile Strength (MPa)	Tensile Fracture Strength (MPa)	Max. Compressive Strength (MPa)	Compressive Yield Strength (MPa)	Max. Flexural Strength (MPa)
PMMA	2.321	2.103	2.45	54.11	53.75	334.37	85.25	113.29
0.05%ZnO	2.353	2.152	2.501	53.57	53.53	382.8	88.03	114.56
0.1%ZnO	2.451	2.202	2.611	55.31	55.30	384.69	90.01	123.02
0.15%ZnO	2.406	2.185	2.722	53.45	53.19	388.53	87.12	125.94
0.20%ZnO	2.382	2.164	2.555	52.72	52.18	345.51	86.24	118.62
0.25%ZnO	2.321	2.121	2.407	51.28	51.28	344.38	84.15	109.11

## Data Availability

The original contributions presented in the study are included in the article; further inquiries can be directed to the corresponding authors.
